# Overexpression of Apolipoprotein A1 in the Lung Abrogates Fibrosis in Experimental Silicosis

**DOI:** 10.1371/journal.pone.0055827

**Published:** 2013-02-08

**Authors:** Eun hee Lee, Eun-ju Lee, Hee jeong Kim, An soo Jang, Eun suk Koh, Soo-taek Uh, Yong hoon Kim, Sung-woo Park, Choon-sik Park

**Affiliations:** 1 Division of Allergy and Respiratory Medicine, Department of Internal Medicine, Soonchunhyang University Bucheon Hospital, Bucheon, Gyeonggi-Do, South Korea; 2 Department of Pathology, Soonchunhyang University Bucheon Hospital, Bucheon, Gyeonggi-Do, South Korea; 3 Division of Allergy and Respiratory Medicine, Soonchunhyang University Seoul Hospital, Seoul, South Korea; 4 Division of Allergy and Respiratory Medicine, Soonchunhyang University Cheonan Hospital, Cheonan, Chungcheongnam-do, South Korea; Helmholtz Zentrum München/Ludwig-Maximilians-University Munich, Germany

## Abstract

The inhalation of silica particles induces silicosis, an inflammatory and fibrotic lung disease characterized by the early accumulation of macrophages and neutrophils in the airspace and subsequent appearance of silicotic nodules as a result of progressive fibrosis. This study evaluated whether apolipoprotein A1 (ApoA1) protects against ongoing fibrosis and promotes the resolution of established experimental lung silicosis. Crystallized silica was intratracheally administered to 6- to 8-week-old transgenic mice expressing human ApoA1 in their alveolar epithelial cells (day 0). ApoA1 was overexpressed beginning on day 7 (ApoA1_D7 group) or day 15 (ApoA1_D15 group). The mice were sacrificed on day 30 for an evaluation of lung histology; the measurement of collagen, transforming growth factor-b1 and lipoxin A4; and a TUNEL assay for apoptotic cells. The ApoA1_D7 and D15 groups showed significant reductions in the silica-induced increase in inflammatory cells, silicotic nodule area, and collagen deposition compared with the silica-treated ApoA1 non-overexpressing mice. The level of transforming growth factor-b1 decreased in the bronchoalveolar lavage fluid, whereas lipoxin A4 was increased in the ApoA1_D7 and D15 groups compared with the silica-treated ApoA1 non-overexpressing mice. The silica-induced increase in the number of apoptotic cells was significantly reduced in the lungs of mice overexpressing ApoA1. Overexpression of ApoA1 decreased silica-induced lung inflammation and fibrotic nodule formation. The restoration of lipoxin A4 may contribute to the protective effect of ApoA1 overexpression against silica-induced lung fibrosis.

## Introduction

Apolipoprotein A1 (ApoA1), the major component of high-density lipoprotein, plays an important role in reverse cholesterol transport by extracting cholesterol and phospholipids from various cells, including lung cells, and transferring them to the liver. In addition to cholesterol efflux, ApoA1 possesses anti-inflammatory and antioxidative properties, and ApoA1 mimetics are an effective treatment for atherosclerosis and several inflammatory disorders in animal models [1], [2], [3]. Using the lung disease model, it has been reported that treatment with ApoA1 mimetics attenuated allergen-induced airway inflammation in murine models of asthma by decreasing oxidative stress [4]. Recently, we reported that ApoA1 is expressed in the lung epithelium, that lung ApoA1 levels were reduced in patients with idiopathic pulmonary fibrosis, and intranasal treatment with ApoA1 significantly attenuated experimental bleomycin-induced lung injury and fibrosis [5]. However, it is unclear whether ApoA1 administration after an injury can reduce established pulmonary fibrosis. Slowly progressive models of fibrosis are generally used to investigate this issue because the disease resolution observed in the bleomycin model does not mimic permanent human fibrosis [6], [7], [8].

Chronic occupational or environmental respiratory exposure to crystalline silica causes the accumulation and activation of inflammatory cells in the lung, leading to tissue damage. The persistence of tissue damage and abnormal repair ultimately leads to fibrosis and a variety of chronic pulmonary diseases such as silicosis [1]. Experimental silicosis is a useful model for exploring the mechanisms and potential therapies in persistent pulmonary fibrosis [9], [10]. Alveolar macrophages and pro-inflammatory cytokines such as tumor necrosis factor (TNF) - α and interleukin (IL)-1β produced by these cells are important in the early inflammatory response after exposure to silica. At a later stage, progressive fibrosis with silicotic nodules and emphysematous changes is observed [11], [12]. The silica mouse model may be suitable as a chronic fibrosis model for investigating the efficacy of ApoA1 in preventing ongoing lung fibrosis or treating established fibrosis. The long-term therapeutic effect of ApoA1 could be assessed by repeated administration via the intranasal route; however, this method has technical limitations such as uneven distribution of ApoA1 and wide variations in delivery into the small airways and alveolar space in mice. To overcome these limitations, we generated ApoA1 transgenic mice, in which the timing of ApoA1 overexpression in the alveolar epithelium can be controlled.

To overcome these limitations, we generated transgenic mice, ApoA1 is overexpressed at endogenous site which is in the alveolar epithelium and the timing of expression can be controlled. The present study, which investigated the therapeutic impact of ApoA1 on silica-induced experimental lung fibrosis, shows that overexpression of ApoA1 diminished the development of lung fibrosis and promoted the resolution of established fibrosis. Some of the results from the present study have been previously reported in abstract form [13].

## Materials and Methods

### Generation of ApoA1 Transgenic Mice and Silica-induced Pulmonary Fibrosis

Inducible human ApoA1 (hApoA1) transgenic mice were produced by the co-injection of *SP-C-rtTA*-hGH (a gift from Dr. Jeffery Whitsett, Cincinnati Children’s Hospital Medical Center, Cincinnati, OH, USA) and pTRE-Tight-ApoA1 into C57BL/6 blastocysts (Orient Bio Inc., Charles River Technology, Sungnam, Korea) as described previously [14]. C57BL/6-Tg (UBC-GFP)30Scha/J mice, 6 to 8 weeks old, expressing enhanced green fluorescent protein (GFP) under the control of the human ubiquitin C promoter were purchased from the Jackson Laboratory (Bar Harbor, ME, USA). The Committee on Animal Research at Soonchunhyang University hospital approved the use of mice for these experiments (SCHBC_Animal_201209). The ApoA1 transgenic mice were given drinking water containing doxycycline (50 mg/mL; Sigma-Aldrich, St. Louis, MO, USA) to induce transgene expression.

On day 0, the transgenic mice received 20 mg of sterile silica crystals (median diameter, 1–5 µm; Sigma-Aldrich) in endotoxin-free water in a total volume of 100 µL by intratracheal delivery. The ApoA1 transgenic male mice (6 to 8 weeks old) were randomly assigned to three groups (*n* = 8/group). Two groups received drinking water containing doxycycline (50 mg/mL), beginning on day 7 (ApoA1_D7 group) or day 15 (ApoA1_D15 group) after silica delivery and continuing until the mice were sacrificed on day 30. A third group of silica-treated transgenic mice (Silica group) received endotoxin-free water containing no doxycycline until they were sacrificed on day 7, 15, or 30. As a control, ApoA1 transgenic mice were treated with phosphate-buffered saline (PBS; 100 µL, intratracheally) on day 0 and housed with or without doxycycline-containing water (50 mg/mL) until day 30 (defined as the ApoA1 or PBS group). To determine whether doxycycline had an anti-fibrotic effect, UBC-GFP transgenic mice were administered silica intratracheally and given drinking water with or without doxycycline (50 mg/mL) from day 0 until they were sacrificed on day 15 or 30. At the end of the experimental period, bronchoalveolar lavage (BAL) was performed as described previously [5].

### RNA Extraction and RT-PCR Amplification of hApoA1, IL-1β, KC, TNF-α, MCP-1 and MIP-2

Total RNA was isolated from mouse lung tissue using TRI REAGENT (Molecular Research Center, Cincinnati, OH, USA) [15] and treated with DNase I (10,000 U/mL) (Stratagene, La Jolla, CA, USA) to remove any contaminated DNA. cDNA was synthesized from 3 µg of total RNA using SuperScript II Reverse Transcriptase (Invitrogen, Grand Island, New York, USA) in a 20-µL reaction including 0.5 mM dNTPs, 2.5 mM MgCl_2_, 5 mM DTT, random hexamers (50 µg/µL), and SuperScript RT (200 U/µL) at 42°C for 50 min, followed by heat inactivation at 70°C for 15 min. Real-time PCR was performed in a 20-µL reaction with 3-µg cDNA, 1 µL of each primer (10 pM), and 10-µL SYBR Green Master Mix using an ABI7500 (Applied Biosystems, Foster City, CA, USA). PCR conditions were as follows; denaturation at 95°C for 10 min and 40 cycles with denaturation at 95°C for 15 s, 60°C for 1 min. The following primers were used for the amplification: hApoA1 sense 5′-ACC ACG CCA AGG CCA CCG AG-3′ and hApoA1 antisense 5′-CTC GAG AGC GCT CAG GAA GCT-3′, mouse ApoA1 sense 5′-CCT AGA GGA AGT GAA ACA G-3′ and mouse ApoA1 antisense 5′-AAG GTA GGG TTG CTC TTG A-3′, GAPDH sense 5′-TGC TGA GTA TGT CGT GGA GTC TA-3′ and GAPDH antisense 5′-AGT GGG AGT TGC TGT TGA AGT CG-3′, IL-1β sense 5′-GTT GAC GGA CCC CAA AAG-3′ and IL-1β antisense 5′-GTG CTG CTG CGA GAT TTG-3′, KC sense 5′-AAA AGG TGT CCC CAA GTA-3′ and KC antisense 5′-AAG CAG AAC TGA ACT ACC ATC G-3′, TNF-α sense 5′-GGG TGT CAA CAG TTA CTA CCC A-3′ and TNF-α antisense 5′-GCT GCA CAT AAA CGG TCT GC-3′, MCP-1 sense 5′-CTT CTG GGC CTG CTG TTC A-3′ and MCP-1 antisense 5′-CCA GCC TAC TCA TTG GGA TCA-3′, MIP-2 sense 5′-CCA CTC TCA AGG GCG GTC AA-3′ and MIP-2 antisense 5′-CCC CTT ATC CCC AGT CTC TTT CAC-3′.

### Histological Assays

A portion of the left lung was fixed in 4% buffered paraformaldehyde and embedded in paraffin. The tissue was cut into 4-µm-thick slices and stained using hematoxylin and eosin or Masson’s trichrome stain. The right lung was quick-frozen by immersion in liquid nitrogen and kept for RNA or protein extraction. The tissue contents were examined by two investigators who were blinded to the origin of the material using light and polarizing microscopy. The area occupied by silicotic nodules and the number of silica particles in the lung were determined using the point-counting technique across 20 random non-coincident microscopic fields at magnifications of ×200 and ×100, respectively, as described previously with some modification [16], [17], [18].

### Collagen Assay

The total amount of soluble collagen in the lung was determined using a Sircol Collagen Assay Kit (Biocolor Ltd., Belfast, Northern Ireland, UK) according to the manufacturer’s instructions [5].

### Terminal Deoxynucleotidyltransferase dUTP Nick End-labeling (TUNEL) Assay

Paraffin-embedded lung tissues from control and ApoA1-expressing mice treated with silica (*n* = 8 mice/group) were labeled using a TUNEL assay kit (Roche Diagnostics, Basel, Switzerland). The numbers of TUNEL-positive (apoptotic) cells on three sections per mouse were counted under a fluorescence microscope at ×400 (Carl Zeiss Microsystems, Thornwood, NY, USA) as described previously [19].

### Measurement of TGF-b1, Lipoxin A4 (LXA4) and hApoA1 in the Lung

The extracted lung tissues were homogenized in a protein lysis solution containing 50 mM Tris-HCl (pH 7.4), 1% NP-40, 50 mM NaCl, 0.5 mM ethylene diamine tetraacetic acid (EDTA), and 100 mM phenylmethylsulfonyl fluoride (PMSF) in distilled water, incubated on ice for 30 min and centrifuged at 14000 rpm for 15 min. The supernatant was collected and used as sample lysates. TGF-b1 and LXA4 from lung lysates and BAL fluid were measured by enzyme-linked immunosorbent assay (ELISA) using kits specific for active TGF-b1 (Promega, Madison, WI, USA) and LXA4 (Neogen, Lexington, KY, USA), according to the manufacturers’ instructions. hApoA1 levels from BAL fluids were measured by ELISA (Abnova, Buckingham, UK).

### Immunoblotting and Immunofluorescence Staining

ApoA1 expression was analyzed by immunoblotting and immunohistochemical staining with goat anti-ApoA1 polyclonal antibodies. The protein samples were fractionated by 12.5% SDS-PAGE and transferred to nitrocellulose membranes (Amersham Pharmacia Biotech, Inc.). The membranes were incubated in a blocking solution containing a 1∶200 dilution of goat anti-ApoA1 polyclonal antibodies (Santa Cruz Biotechnology, Inc., Santa Cruz, CA, USA) and then incubated with a blocking solution containing a 1∶5,000 dilution of horseradish peroxidase-conjugated polyclonal anti-goat IgG antibodies. For Caspase-3 expression, a 1∶1000 dilution of anti-Caspase-3 polyclonal antibody (Cell Signaling Technology, Beverly, MA, USA) was used. Enhanced chemiluminescence detection was performed according to the manufacturer’s instructions (Boehringer Mannheim, Mannheim, Germany). The relative abundance of protein was determined by quantitative densitometry using Image J software (NIH, Bethesda, MD). All Western Blot densitometry data were normalized to β-actin.

For the immunohistochemical analysis of ApoA1, mouse lung tissue was incubated at 4°C overnight with goat anti-ApoA1 polyclonal antibodies (1∶100 dilution; Abcam) and rabbit anti-prosurfactant protein C (proSP-C) polyclonal antibodies (1∶100 dilution; Chemicon-Millipore, Billerica, MA, USA). Fluorescein isothiocyanate-conjugated donkey anti-goat antibodies (1∶1,000; Santa Cruz Biotechnology, Inc.) and goat anti-rabbit IgG-PE (1∶1,000; Santa Cruz Biotechnology, Inc.) were used as secondary antibodies for the localization of ApoA1 and SPC in mouse lung.

For localization of apoptotic cells, antibodies against proSP-C (1∶100; Chemicon-Millipore, Billerica, MA, USA), anti-F4/80 (1∶100; Ebioscience Inc., San Diego, CA, USA) were used to identify alveolar epithelial cells and macrophages, respectively. Goat anti-rabbit IgG-PE (1∶500; Santa Cruz Biotechnology, Inc.) and rabbit anti-mouse IgG-PE (1∶500; Santa Cruz Biotechnology, Inc) were used as secondary antibodies.

### Statistical Analysis

All data are expressed as the means ± standard error of the mean. Data were analyzed using the Kruskal-Wallis test followed by the Mann-Whitney U test with the Bonferroni correction for intergroup comparisons. *p*<0.05 was deemed to indicate statistical significance.

## Results

### Doxycycline-induced Overexpression of hApoA1 in Transgenic Mice

**Figure 1:**
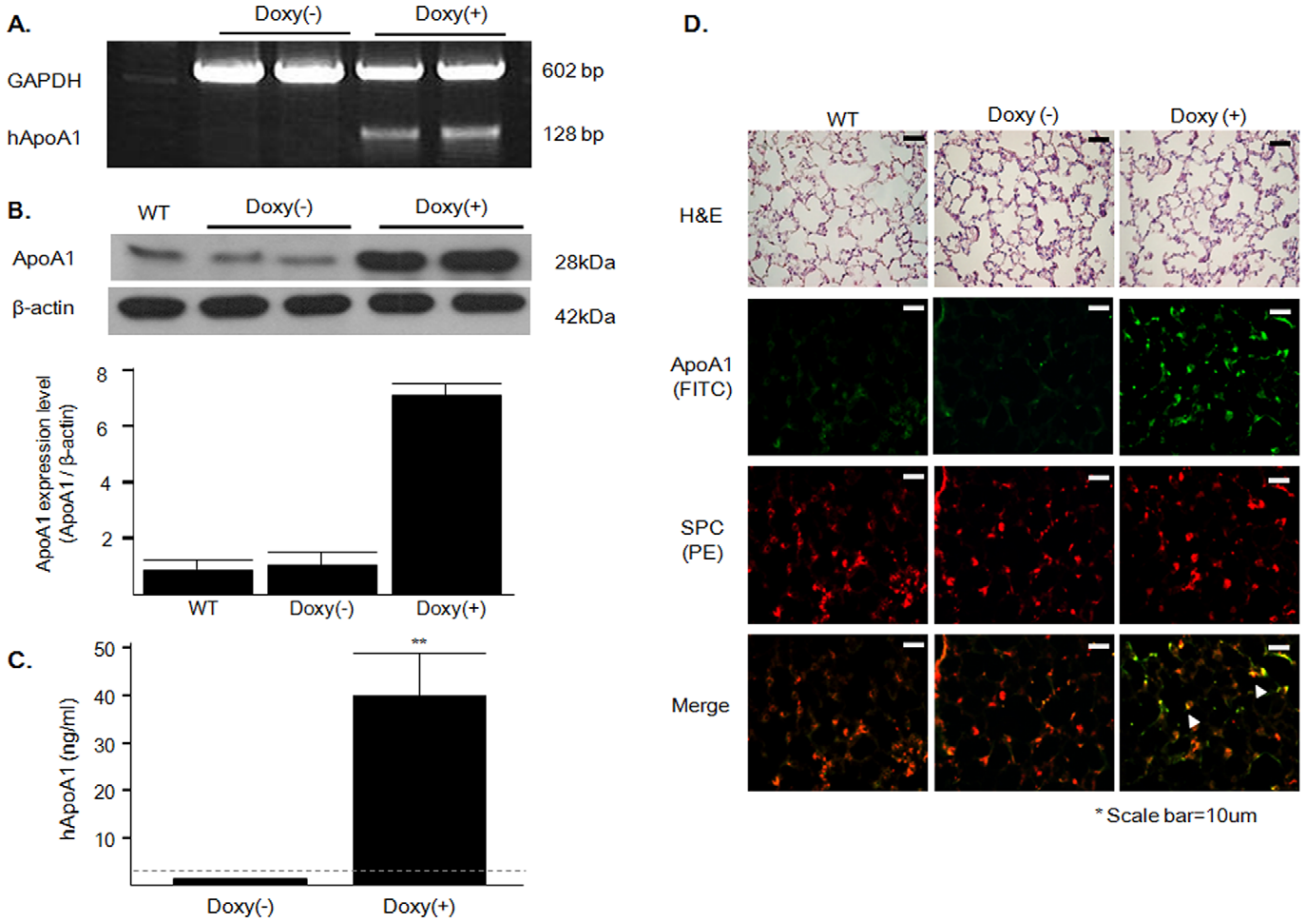
Generation of ApoA1 transgenic mice. Overexpression of hApoA1 was induced using doxycycline-containing drinking water (Doxy+). Control mice were offered normal water (Doxy−). (*A)* RT-PCR analysis revealed hApoA1 mRNA only in the lungs of mice treated with doxycycline. (*B)* ApoA1 protein expression was significantly greater in the lungs of doxycycline-treated transgenic mice compared with those of wild-type or transgenic mice not treated with doxycycline (*n* = 6 mice/group). **p<0.01 compared with WT or Doxy(−) mice. (C) Detection of human ApoA1 in BAL fluids. ELISA was performed on the first 1-mL fraction of BAL fluid, with a detection limit of 3.13 ng/mL (dashed line). Values below the detection limit were treated as 0 (*n* = 6 mice/group). **p<0.01 compared with Doxy(−) mice. (D*)* Immunofluorescence analysis showed that ApoA1 was strongly expressed in the alveolar epithelium (white arrow heads) in the doxycycline-treated transgenic mice. Scale bar = 10 µm. H&E, hematoxylin and eosin.

PCR analysis showed that hApoA1 mRNA was expressed in the lungs of the ApoA1 transgenic mice only after doxycycline treatment, suggesting that hApoA1 expression is tightly regulated by doxycycline (Fig. 1A). Although the antibody against hApoA1 also detected mouse ApoA1 owing to sequence similarity, ApoA1 was 6.6-times more strongly expressed in the lungs of the doxycycline-treated transgenic mice compared with transgenic mice that were not treated with doxycycline and wild-type mice (Fig. 1B). Furthermore, there was no difference in ApoA1 expression between the transgenic mice not treated with doxycycline and wild-type mice (Fig. 1B). hApoA1 was detected in the BAL fluid of the doxycycline-treated transgenic mice, but not in those that were not treated with doxycycline (Fig. 1C). We also observed that endogenous ApoA1 mRNA, hApoA1 mRNA and secreted hApoA1 levels were maintained throughout the duration of the experiment (Figure S1). Immunofluorescence analysis revealed strong staining for ApoA1 in the alveolar epithelial cells following treatment with doxycycline (Fig. 1D).

### Effect of Apo A1 Overexpression on Silica-induced Inflammation and Nodule Formation

**Figure 2:**
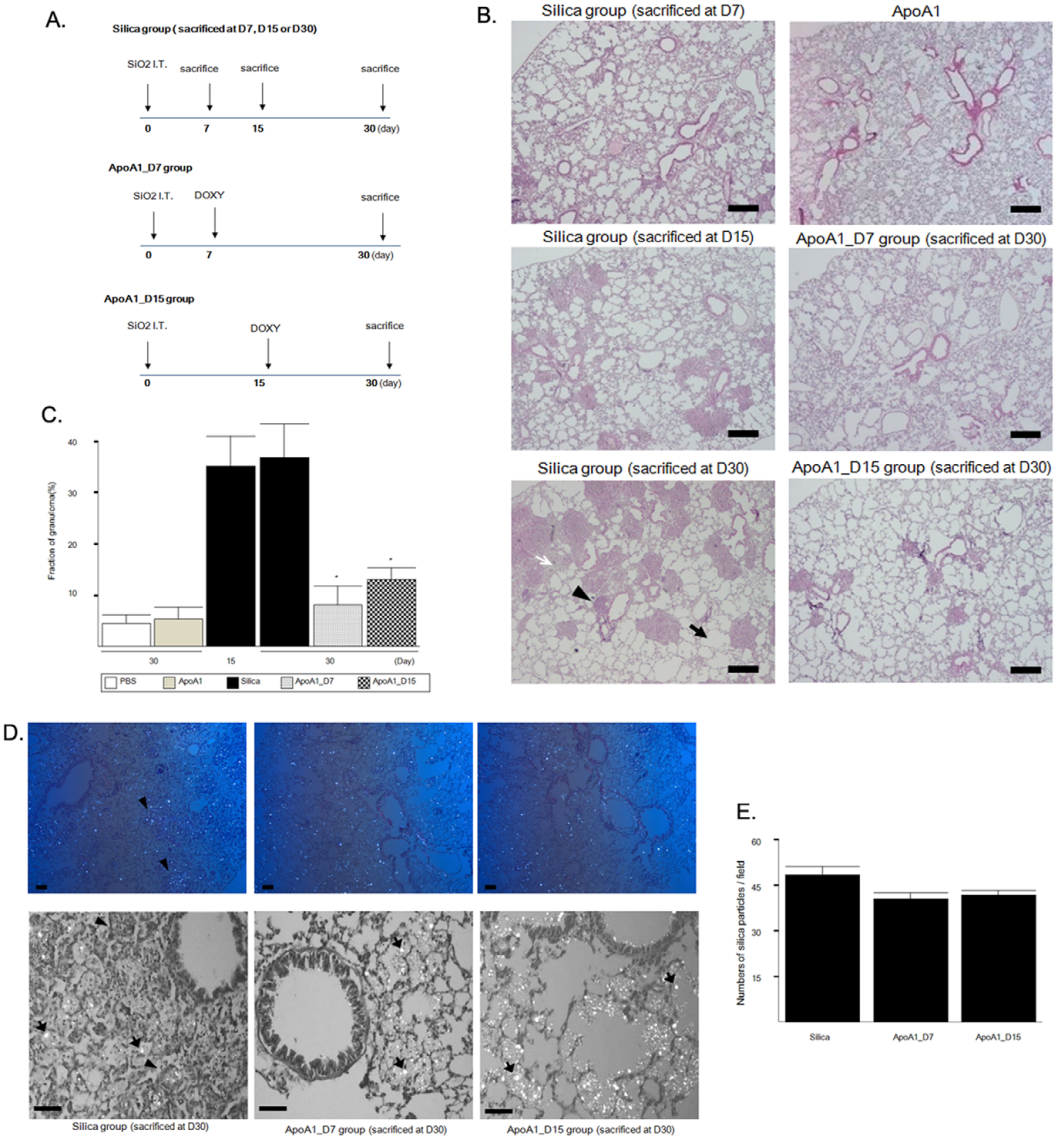
Experimental protocol and histological analysis of lung tissues from ApoA1 transgenic mice. (*A)* Silica was administered via the trachea to ApoA1 transgenic mice (6 to 8 weeks old) on day 0. The mice were given drinking water containing doxycycline to induce hApoA1 overexpression beginning on day 7 (ApoA1_D7 group) or 15 (ApoA1_D15 group) and continuing throughout the experiment. A third group of silica-treated transgenic mice, which received drinking water with no doxycycline during the experiment (Silica group), were sacrificed 7, 15, or 30 days after silica administration. The ApoA1_D7 and D15 mice were sacrificed 30 days after silica administration. (*B)* Hematoxylin and eosin staining of lung sections from the transgenic mice following silica administration and doxycycline treatment on day 7 (ApoA1_D7) or 15 (ApoA1_D15), and in those that received no doxycycline treatment (Silica group). Silica group D7, D15, and D30 correspond to mice sacrificed 7 (D7), 15 (D15), or 30 days (D30) after silica administration, respectively. Scale bar = 100 µm. (*C)* Quantification of the area occupied by silicotic nodules in the lung (*n* = 8/group). **p<0.01 compared with the Silica group (D30). (*D)* Polarizing light microscopic analysis showed silica particles in the alveolar space in the lungs of the Silica, ApoA1_D7, and ApoA1_D15 group mice. Scale bar = 20 µm. (*E)* Numbers of silica particles were observed at ×100 magnification using polarizing microscopy, and the number of silica particles in 20 fields per lung was counted (*n* = 8 mice/group).

**Figure 3:**
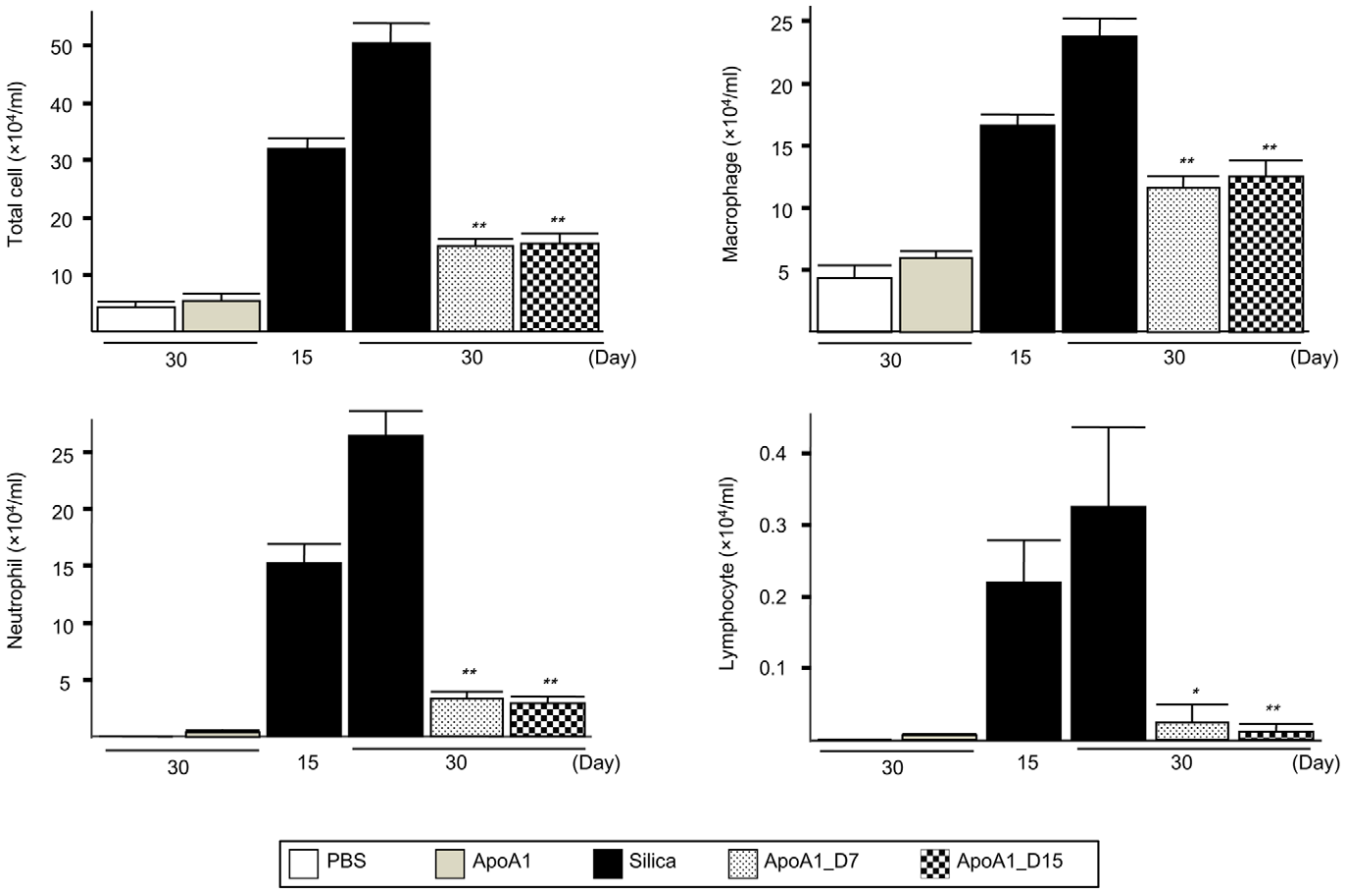
Differential cell counts from the BAL fluid of ApoA1 transgenic mice. **p<0.01 compared with the Silica group (D15 and D30).

Silica is well established as an agent for inducing pulmonary inflammation and fibrosis [20]. Our experimental protocol is shown in Fig. 2A. A histological examination of the Silica group mice on day 30 showed interstitial edema and peribronchial silicotic nodules with extensive accumulation of inflammatory cells, as well as alveolar collapse and emphysema (Fig. 2B). In the ApoA1_D7 and D15 mice, silica-induced inflammation was nearly absent, and the silicotic nodule area was significantly decreased (Fig. 2B, C). Polarizing microscopy revealed that silica particles were present in the nodules of the silica-exposed mice, with a similar number of silica particles present in the alveolar space and walls of the Silica group, ApoA1_D7, and ApoA1_D15 mice (Fig. 2D, E). The total number of inflammatory cells, macrophages, neutrophils, and lymphocytes in the BAL fluid were significantly decreased in the ApoA1_D7 and D15 groups compared with the Silica group, and no significant difference in the number of inflammatory cells was found between the ApoA1_D7 and D15 groups (Fig. 3).

### Effect of ApoA1 Overexpression on Collagen and TGF-b1 in the Lung

**Figure 4:**
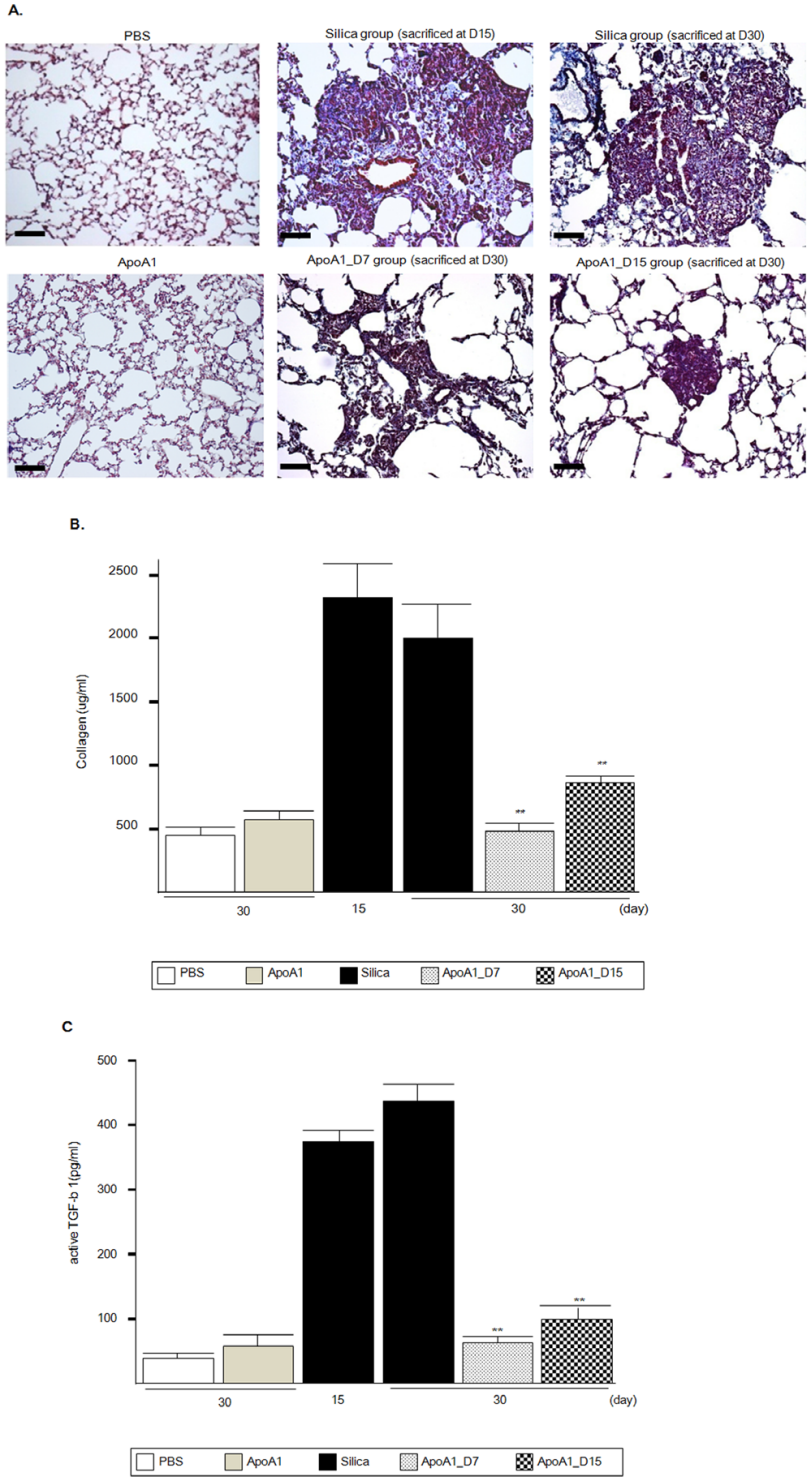
Quantification of the lung collagen and TGF levels in the ApoA1 transgenic mice. (*A)* Masson’s trichrome staining of lung sections. Scale bar = 20 µm. (*B)* Soluble lung collagen was measured using a Sircol assay. **p<0.01 compared with the Silica group (D15 and D30). (*C)* The level of the active TGF-b1 in the lung was measured by ELISA. **p<0.01 compared with the Silica group (D15 and D30).

Masson’s trichrome staining revealed a decrease in collagen deposition in the lungs of the ApoA1_D7 and D15 mice compared with that in the Silica group (Fig. 4A). Lung-soluble collagen was also significantly reduced in the lungs of both groups compared with the Silica group (Fig. 4B). The level of the active form of TGF-b1 in the lung was significantly increased following treatment with silica, but was significantly decreased in the ApoA1_D7 and D15 groups (Fig. 4C).

### Effect of ApoA1 Overexpression on Lipid Mediator of Inflammation

**Figure 5:**
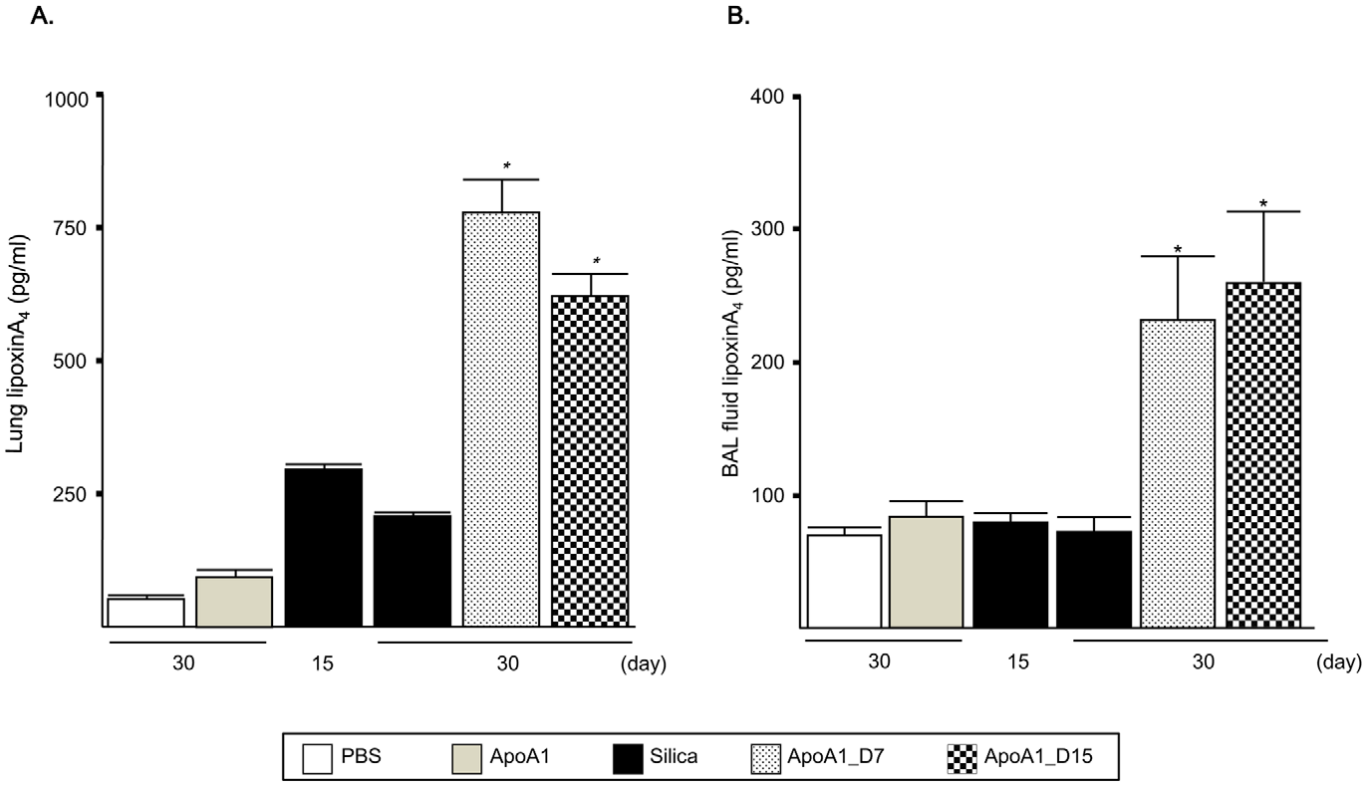
Quantification of LXA4 levels in the lung parenchyma(A) and BAL fluid(B) of the ApoA1 transgenic mice. *p<0.05 compared with the Silica group (D30).

Given the important role of lipid mediators in the initiation, maintenance, and resolution of inflammation, the levels of anti-inflammatory LXA4 were measured in the lungs of silica-treated ApoA1_D7 and D15 mice. ApoA1 overexpression was associated with increased levels of LXA4 in the lung parenchyma and BAL fluid (Fig. 5).

### Effect of ApoA1 on Silica-induced Apoptosis in Mouse Lung

**Figure 6:**
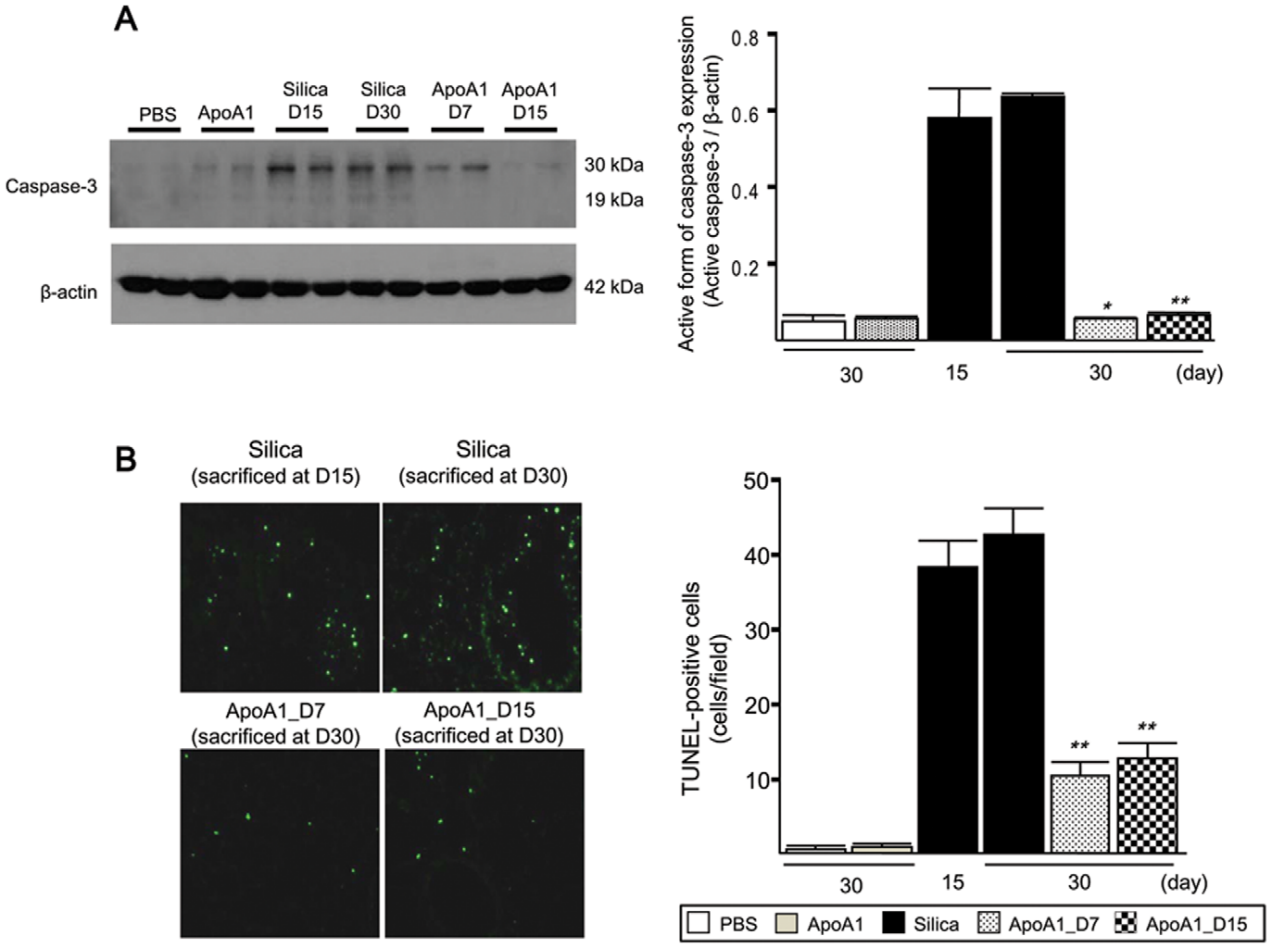
Quantification of silica-induced apoptosis in the lungs of the ApoA1 transgenic mice. (*A)* Immunoblot analysis showed that active form of caspase-3 protein expression was significantly decreased in the ApoA1_D7 and D15 groups compared with the Silica group. **p<0.01 compared with the Silica group (D30). *p<0.05 compared with the Silica group (D30). *(B)* Tissues stained by the TUNEL method were observed at ×100 magnification, and the number of TUNEL-positive cells in a minimum of 20 fields per lung was counted (*n* = 8 mice/group). **p<0.01 compared with the Silica group (D15 and D30).

To determine whether ApoA1 inhibited silica-induced apoptosis, we measured the levels of caspase-3 expression in the lungs. Levels of caspase-3 protein were increased in the Silica group compared with the sham-exposed controls. The levels of active form of caspase-3 protein in the ApoA1_D7 and D15 groups were significantly decreased compared with that in the Silica group (Fig. 6A). Similarly, a TUNEL assay revealed approximately 75% fewer apoptotic cells in the lungs of the ApoA1-overexpressing mice compared with the lungs of the Silica group mice (Fig. 6B). Double-labeled immunofluorescence demonstrated that the majority of apoptotic cells were alveolar epithelial cells and macrophages (Figure S2).

### Effect of ApoA1 on Silica-induced Increased Proinflammatory Mediators in Mouse Lung

**Figure 7:**
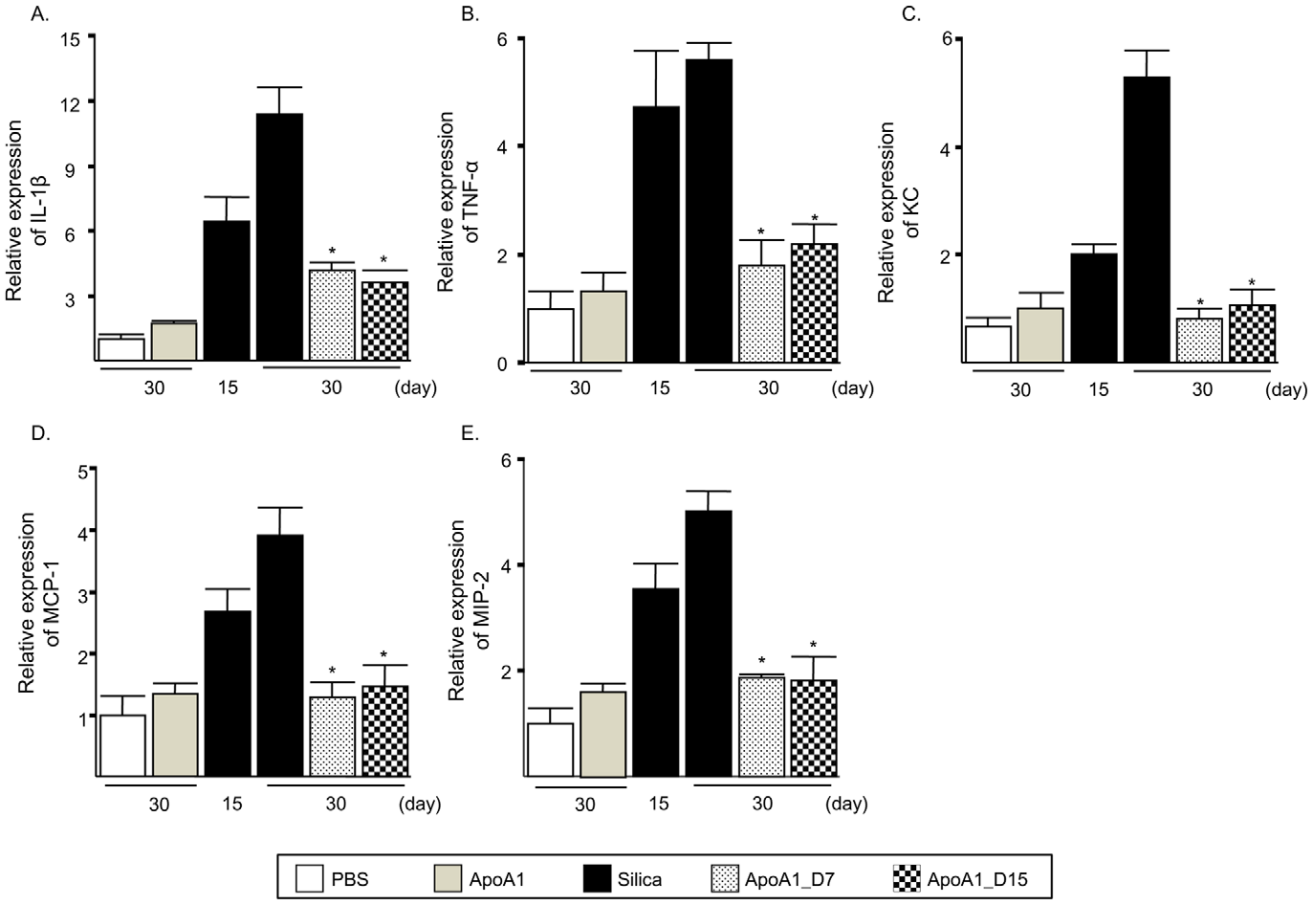
Levels of proinflammatory mediator mRNAs in the lungs of ApoA1 transgenic mice. IL-1β *(A)*, TNF-α *(B)*, KC *(C)*, MCP-1 *(D)* and MIP-2 *(E)* mRNA expression were measured by real-time PCR. *p<0.05 compared with the Silica group (D30).

To determine whether ApoA1 inhibits the increase of the proinflammatory cytokines and chemokines that accumulate from macrophages and neutrophils stimulated by silica, mRNA levels of interleukin-1ß (IL-1ß), tumor necrosis factor-α (TNF-α), monocyte chemotactic protein-1 (MCP-1), macrophage inflammatory protein-2 (MIP-2) and chemokine (C-X-C motif) ligand 1 (CXCL1, synonym KC) in the lungs were measured. All of the measured levels of proinflammatory mediator mRNAs in the ApoA1_D7 and D15 group were significantly decreased compared with those in the Silica group (Fig. 7).

### Effect of Doxycycline on Silica-induced Lung Inflammation and Fibrosis in Mouse Lung

Doxycycline reportedly has an anti-fibrotic effect on bleomycin-induced experimental fibrosis [21]. To determine whether treatment with doxycycline contributed to the anti-fibrotic effect on ApoA1 transgenic mice, silica was administered intratracheally to the UBC-GFP transgenic mice with or without doxycycline-containing water. The histologic findings showed severe peribronchial nodules, inflammatory cell accumulation, and interstitial edema in both the silica-exposed doxycycline mice and those fed drinking water that did not contain doxycycline (Figure S3A). The total numbers of inflammatory cells, macrophages, neutrophils, and lymphocytes in the BAL fluid were not different between the two groups (Figure S3B). Silica induced increased amounts of lung-soluble collagen, and the granuloma fractions were not decreased by doxycycline treatment (Figure S3C, D).

## Discussion

The results of the present study show that the overexpression of hApoA1 in a murine silicosis model, which histologically mimics human silicosis, reduced the area occupied by silicotic nodules, the number of inflammatory cells, and the presence of collagen. The beneficial effect of ApoA1 overexpression was associated with decreases in silica-induced active form of TGF-b1 synthesis and apoptotic activity and an increase in endogenous LXA4 synthesis. The silica treatment increased the number of neutrophils and macrophages in the BAL fluid, which may be the source of the TGF-b1 and other inflammatory mediators. Silica has been shown to induce apoptosis in alveolar macrophages, and this may play an important role in the pathogenesis of silicosis [22], [23].

Our findings showing a decrease in caspase-3 protein expression and the number of TUNEL-positive cells suggest that ApoA1 inhibited silica-induced apoptosis in the lung. In a previous study, we reported that local treatment with ApoA1 was effective against the development of bleomycin-induced lung injury/fibrosis [5]. However, the ability of intratracheal bleomycin to induce experimental lung fibrosis has been reported to be self-limiting after 28 days [6], [8]. Moreover, at 14 days after intratracheal bleomycin administration, when the mice were studied, the induced model might have more closely resembled lung injury than fibrosis. In contrast, because silica is not readily cleared from the lung, the pro-fibrotic stimulus is more persistent and fibrosis is easily identified as fibrotic nodules in areas of silica deposition.

Studies using mouse models have shown that the initial inflammatory reaction occurs within the first seven days after crystalline silica delivery via the intratracheal route [24], [25]. Some studies using a mouse model have shown the development of fibrosis within the first month after exposure [26], [27], while others have detected silicotic nodules, which represent fibrosis, at 15 days after silica administration [28], [29]. Doxycycline controlled the expression of hApoA1 protein in the alveolar epithelial cells of our transgenic mice, allowing us to look at chronic treatment with ApoA1 starting at different time points after silica exposure. In the present study, lung inflammation and small silicotic nodules were observed on day 7, and large silicotic nodules had developed by day 15 after intratracheal silica administration (Fig. 2B). Thus, by initiating doxycycline treatment at 7 days after silica administration (ApoA1_D7 group), we examined the therapeutic effect of AopA1 overexpression on inflammation and early fibrosis, and its effectiveness in established fibrosis was studied by starting doxycycline treatment at 15 days after silica administration (ApoA1_D15 group). The ApoA1_D7 and D15 groups showed decreased levels of lung inflammation and silicotic nodule formation compared with the Silica group. As silicotic nodules were well established by day 15 in the Silica group mice, the decreases in the silicotic nodule fraction and collagen deposition in the ApoA1_D15 mice indicate that ApoA1 overexpression had a therapeutic effect on established silica-induced lung inflammation and fibrosis in the present study. Given a previous report showing that doxycycline suppressed fibrosis in a beomycin-induced lung injury/fibrosis model through the inhibition of MMP production [21], we used UBC-GFP transgenic mice to study the effect of doxycycline on silica-induced lung fibrosis. The lungs of the silica-exposed doxycycline-treated mice showed similar levels of inflammation, granuloma formation, and collagen deposition compared with the distilled water-treated mice in both the D15 and D30 groups (Figure S3). These data suggest that the doxycycline that was used for the overexpression of hApoA1 did not contribute to the inhibition of lung inflammation and fibrosis in our silica-induced lung fibrosis model.

Silica deposits in the lung cause a persistent, toxic, and inflammatory response, including the alveolar accumulation of macrophages and neutrophils [26]. We found that in the ApoA1_D7 and D15 groups, lung mRNA levels of the pro-inflammatory mediators KC, MIP-2 and MCP-1, which are known to recruit neutrophils and macrophages to inflamed sites, were significantly decreased compared to those of the silica group (Fig. 7). We also observed abundant silica particles without inflammatory cell accumulation in the airspaces of the ApoA1_D7 and D15 mice, indicating that ApoA1 overexpression in the airspace reduced silica-induced inflammatory cell accumulation and silica nodule formation, despite the presence of silica particles in the lung. We speculate that ApoA1 inhibited the accumulation and migration of alveolar inflammatory cells, particularly macrophages and neutrophils. A decrease in the number or activity of inflammatory cells could account for the observed reduction in the level of TGF-b1, which can induce mesenchymal cell proliferation and extracellular collagen deposition in fibrosis [30]. There are other possible mechanisms for the protective and therapeutic effect of ApoA1 including, an anti-oxidative effect [4] against silica-induced oxidant stress, a direct anti-inflammatory effect of ApoA1 on silica particles since ApoA1 is known to bind to silica particles leading to repression of the inflammatory cytokine and chemokine responses [31]. However, these findings should be validated in further studies.

Lipid mediators participate in the resolution of inflammation and the return to homeostasis in inflamed tissues [32]. LXA4 is a potent anti-inflammatory lipid mediator [33]. In the present study, LXA4 levels were increased in the lung and BAL fluid of ApoA1_D15 mice, suggesting that ApoA1 regulates inflammation-related lipid mediators. The reduction in established silicosis in the lungs of the ApoA1_D7 and D15 mice may be explained in part by the increase in LXA4 activity. Lipoxins are biologically active eicosanoids with anti-inflammatory properties that are produced by lipoxygenases at sites of inflammation [33], [34]. LXA4 has been reported to inhibit leukocyte trafficking by attenuating the release of pro-inflammatory cytokines and chemokines such as TNF-α, IL-8, and macrophage inflammatory protein-2 by inflammatory cells [35], [36], [37]. In addition to its anti-inflammatory activity, LXA4 stimulates neutrophils to phagocytize apoptotic cells without the release of pro-inflammatory cytokines [38], [39], thereby leading to the resolution of inflammation. Exogenous resolvin E1 was recently shown to regulate LXA4 in resolving established airway inflammation in an ovalbumin-challenged mouse asthma model [40]. Recently, Borgeson *et al*. reported that LXA4 have anti-fibrotic effect on renal fibrosis [41]. However, no therapeutic strategies have been proven to attenuate established lung fibrosis to date. To our knowledge, the present study is the first to show a therapeutic effect on established experimental lung fibrosis. The precise mechanisms mediating the beneficial effects of ApoA1 on silica-induced fibrosis remain to be elucidated in future studies.

In summary, the findings of the present study indicate that local treatment with human ApoA1 may reduce both early and established lung inflammation and fibrosis by inhibiting the production of TGF-b1, reducing the number of apoptotic cells and increasing the level of the anti-inflammatory lipid mediator LXA4. ApoA1 appears to be a promising therapeutic agent for the treatment of established lung fibrosis.

## Supporting Information

Figure S1 **Time courses of endogenous ApoA1** ***(A),*** **hApoA1 mRNA expression** ***(B)*** **and secreted hApoA1** ***(C)*** **levels in the lungs of ApoA1 transgenic mice determined by real-time PCR and ELISA, respectively.** ELISA was performed on the first 1-mL fraction of BAL fluid, with a detection limit of 3.13 ng/mL (dashed line).
(TIF)

Figure S2 **Localization of apoptotic cells in the mouse lung detected by double-labeled immunofluororescence.** Pro-surfactant C (Pro-SPC) and TUNEL stain and merged image (white arrows, double positive cells; ×100 original magnification). F4/80 and TUNEL stain and merged image (white arrows, double-positive cells; ×100 original magnification).
(TIF)

Figure S3 **Histological analysis and quantification of lung inflammation and fibrosis in silica administered intratracheally to UBC-GFP transgenic mice that received doxycycline or distilled water.** *(A*) Hematoxylin and eosin staining of lung sections. Scale bar = 20 µm *(B*) Differential cell counts from BAL fluid. (*C)* Quantification of the area occupied by silicotic nodules in the lung (*n* = 6/group). *(D)* Quantification of the soluble lung collagen amounts using a Sircol assay.
(TIF)
