# Supplementary figures and images for: Overexpression of Apolipoprotein A1 in the Lung Abrogates Fibrosis in Experimental Silicosis
Source: PLoS One. 2013 Feb 8;8(2):e55827. doi: 10.1371/journal.pone.0055827 (PMC3568133; doi:10.1371/journal.pone.0055827)

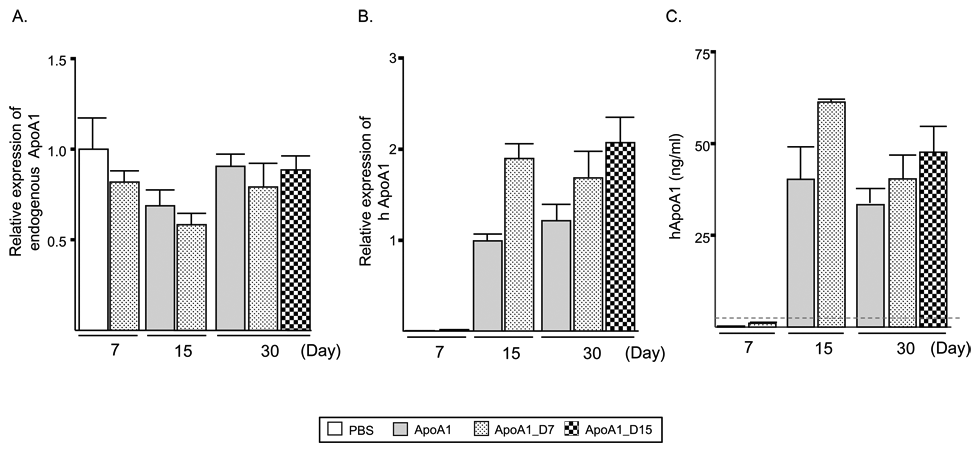

Supplement: Figure S1 — Time courses of endogenous ApoA1 (A), hApoA1 mRNA expression (B) and secreted hApoA1 (C) levels in the lungs of ApoA1 transgenic mice determined by real-time PCR and ELISA, respectively. ELISA was performed on the first 1-mL fraction of BAL fluid, with a detection limit of 3.13 ng/mL (dashed line). (TIF) [file pone.0055827.s001.tif]

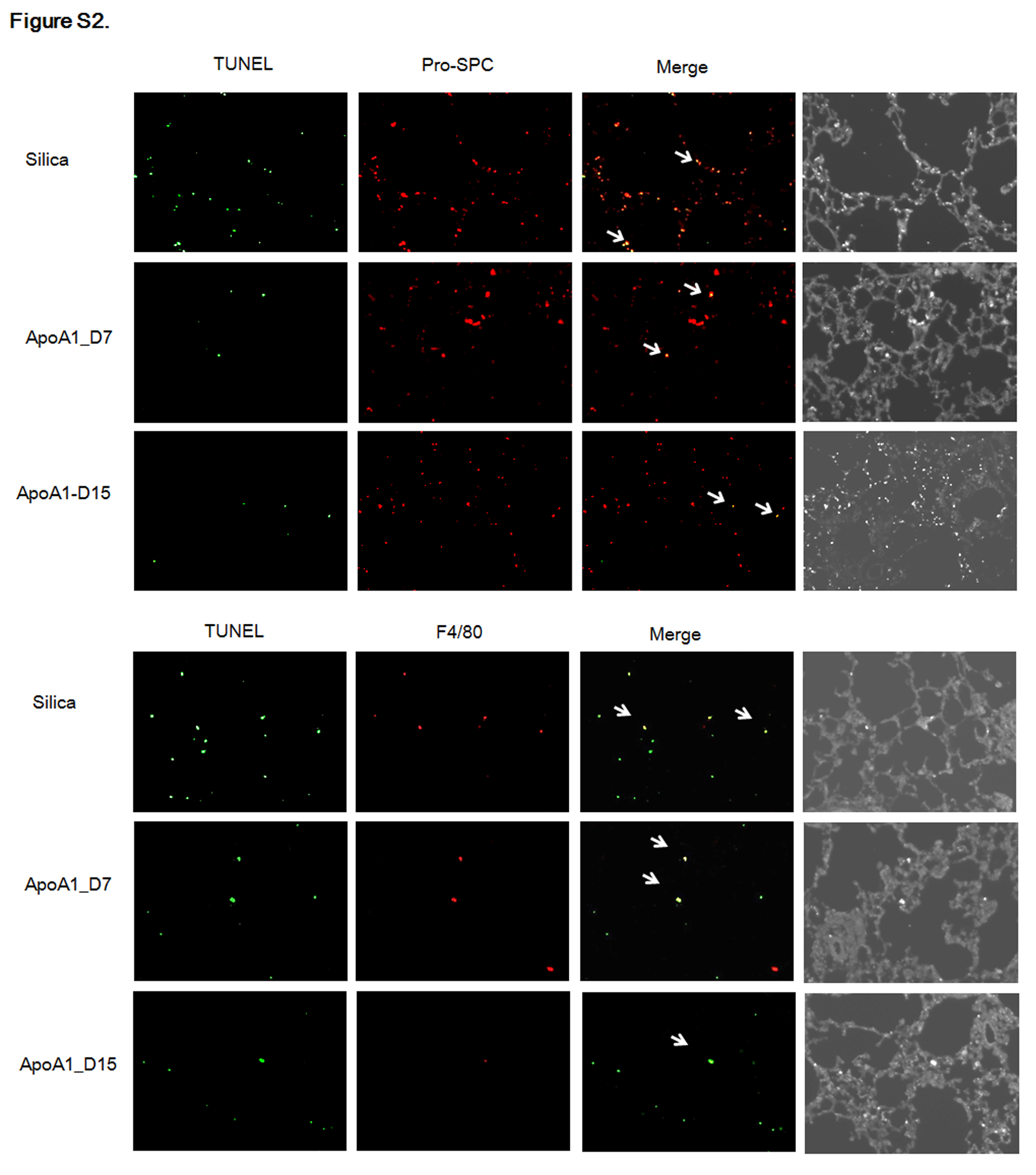

Supplement: Figure S2 — Localization of apoptotic cells in the mouse lung detected by double-labeled immunofluororescence. Pro-surfactant C (Pro-SPC) and TUNEL stain and merged image (white arrows, double positive cells; ×100 original magnification). F4/80 and TUNEL stain and merged image (white arrows, double-positive cells; ×100 original magnification). (TIF) [file pone.0055827.s002.tif]

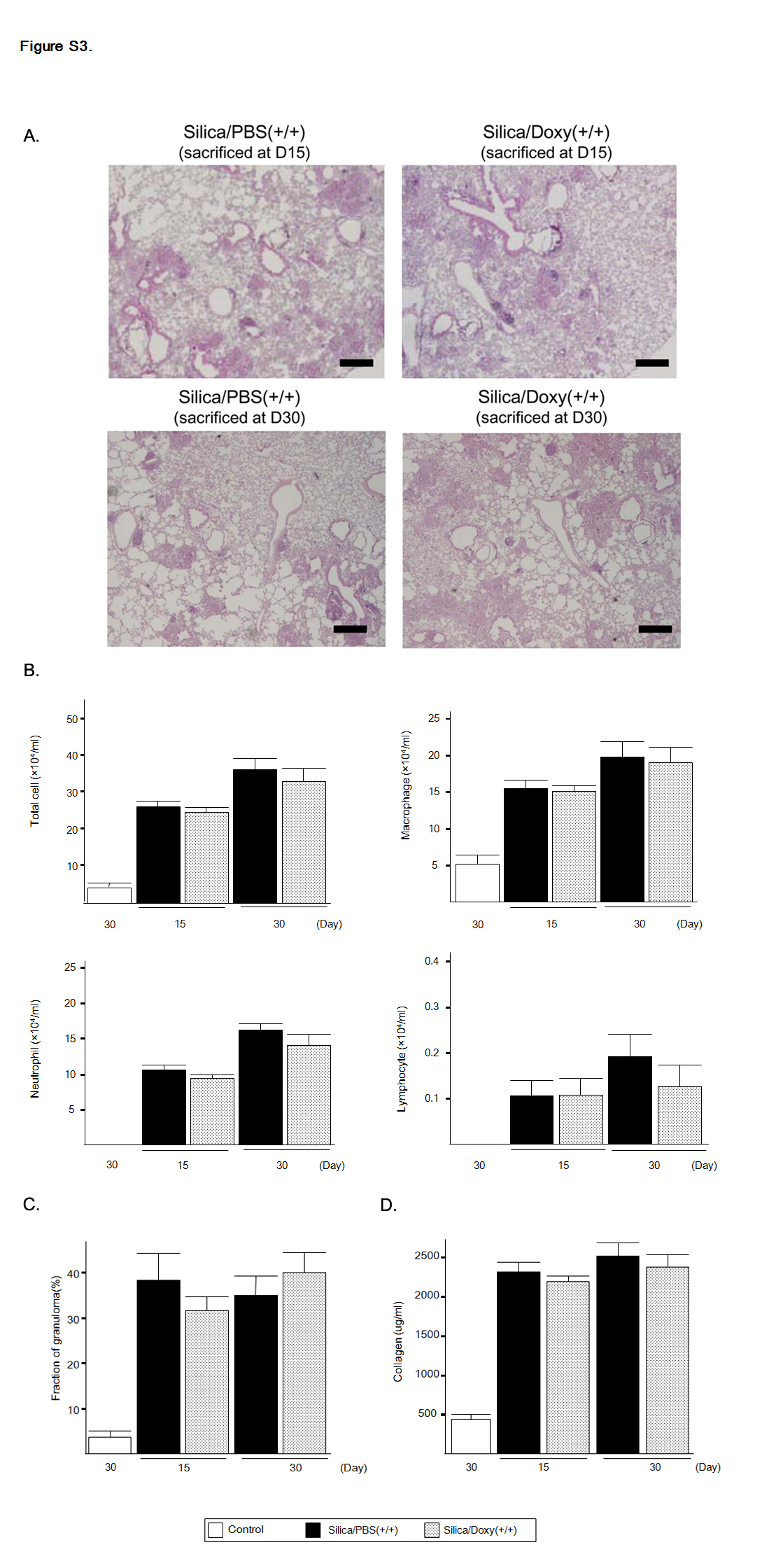

Supplement: Figure S3 — Histological analysis and quantification of lung inflammation and fibrosis in silica administered intratracheally to UBC-GFP transgenic mice that received doxycycline or distilled water. (A) Hematoxylin and eosin staining of lung sections. Scale bar = 20 µm (B) Differential cell counts from BAL fluid. (C) Quantification of the area occupied by silicotic nodules in the lung (n = 6/group). (D) Quantification of the soluble lung collagen amounts using a Sircol assay. (TIF) [file pone.0055827.s003.tif]
